# FOXO1 forkhead domain mutants in B-cell lymphoma lack transcriptional activity

**DOI:** 10.1038/s41598-022-05334-4

**Published:** 2022-01-25

**Authors:** Ariane Sablon, Emeline Bollaert, Constance Pirson, Amélie I. Velghe, Jean-Baptiste Demoulin

**Affiliations:** Experimental Medicine Unit, De Duve Institute, Université Catholique de Louvain, Avenue Hippocrate 75, B1.74.05, 1200 Brussels, Belgium

**Keywords:** Lymphoma, Transcription

## Abstract

Somatic point mutations of the FOXO1 transcription factor were reported in non-Hodgkin lymphoma including diffuse large B-cell lymphoma, follicular lymphoma and Burkitt lymphoma. These alterations were associated with a poor prognosis and resistance to therapy. Nearly all amino acid substitutions are localized in two major clusters, affecting either the N-terminal region (Nt mutations) or the forkhead DNA-binding domain (DBD mutations). While recent studies have focused on Nt mutations, we characterized FOXO1 DBD mutants. We analyzed their transcriptional activity, DNA binding, phosphorylation and protein–protein interaction. The majority of DBD mutants showed a decrease in activity and DNA binding, while preserving AKT phosphorylation and interaction with the cytoplasmic ATG7 protein. In addition, we investigated the importance of conserved residues of the α-helix 3 of the DBD. Amino acids I213, R214, H215 and L217 appeared to be crucial for FOXO1 activity. Our data underlined the key role of multiple amino-acid residues of the forkhead domain in FOXO1 transcriptional activity and revealed a new type of FOXO1 loss-of-function mutations in B-cell lymphoma.

## Introduction

FOXOs are widely expressed transcription factors showing tumor suppressor functions by regulating a plethora of target genes to slow down the cell cycle, induce apoptosis, promote DNA repair and prevent oxidative stress. FOXO1 is inhibited by the oncogenic PI3K-AKT pathway. AKT directly phosphorylates FOXO1 on three different sites (Thr24, Ser256, Ser319), leading to its transcriptional inactivation by nuclear export^1^. In line with these cellular activities and regulations, FOXO1 loss-of-function alterations have been reported in numerous cancer types, including classical Hodgkin lymphoma^2^. However, the well-defined tumor suppressor role of FOXO1 has been challenged by recent studies that demonstrated cancer-promoting functions of FOXO in different cellular context^3–5^.

Multiple next-generation sequencing analyses identified FOXO1 point mutations in non-Hodgkin lymphoma: around 10% in diffuse large B-cell lymphoma (DLBCL)^6,7^, 15% in follicular lymphoma^8^ and 12 to 29% in sporadic Burkitt lymphoma^9,10^. Trinh et al*.* showed that *FOXO1* gene alterations were associated with resistance to treatment (R-CHOP) and poor prognosis in DLBCL^11^. On the one hand, about half of FOXO1 amino acid substitutions clustered in the *N*-terminal region (Nt mutations)^11^. The recurrent M1? and T24I mutants were characterized as gain of function by escaping the PI3K-AKT regulation, leading to nuclear sequestration. Interestingly, several studies suggested pro-proliferative effects of wild-type FOXO1 and Nt mutants on lymphoma cells^10,12,13^. Others also highlighted their central role in the germinal center program^14,15^. On the other hand, the other half of FOXO1 mutations occurs in the forkhead DNA-binding domain (DBD mutations)^11^. The role of FOXO1 DBD mutants has not been defined yet.

In this study, we clarified the impact of DBD mutations on FOXO1 transcriptional activity, DNA binding, phosphorylation and protein–protein interaction. We then investigated the importance of the α-helix 3 of the forkhead domain by studying the consequence of some amino acid substitutions on transcriptional activity. Together, our results revealed the existence of FOXO1 loss-of-function mutations in non-Hodgkin lymphoma, suggesting that FOXO1 plays a complex role in this disease.

## Results

### FOXO1 mutations affect transcriptional activity and DNA binding

**Figure 1:**
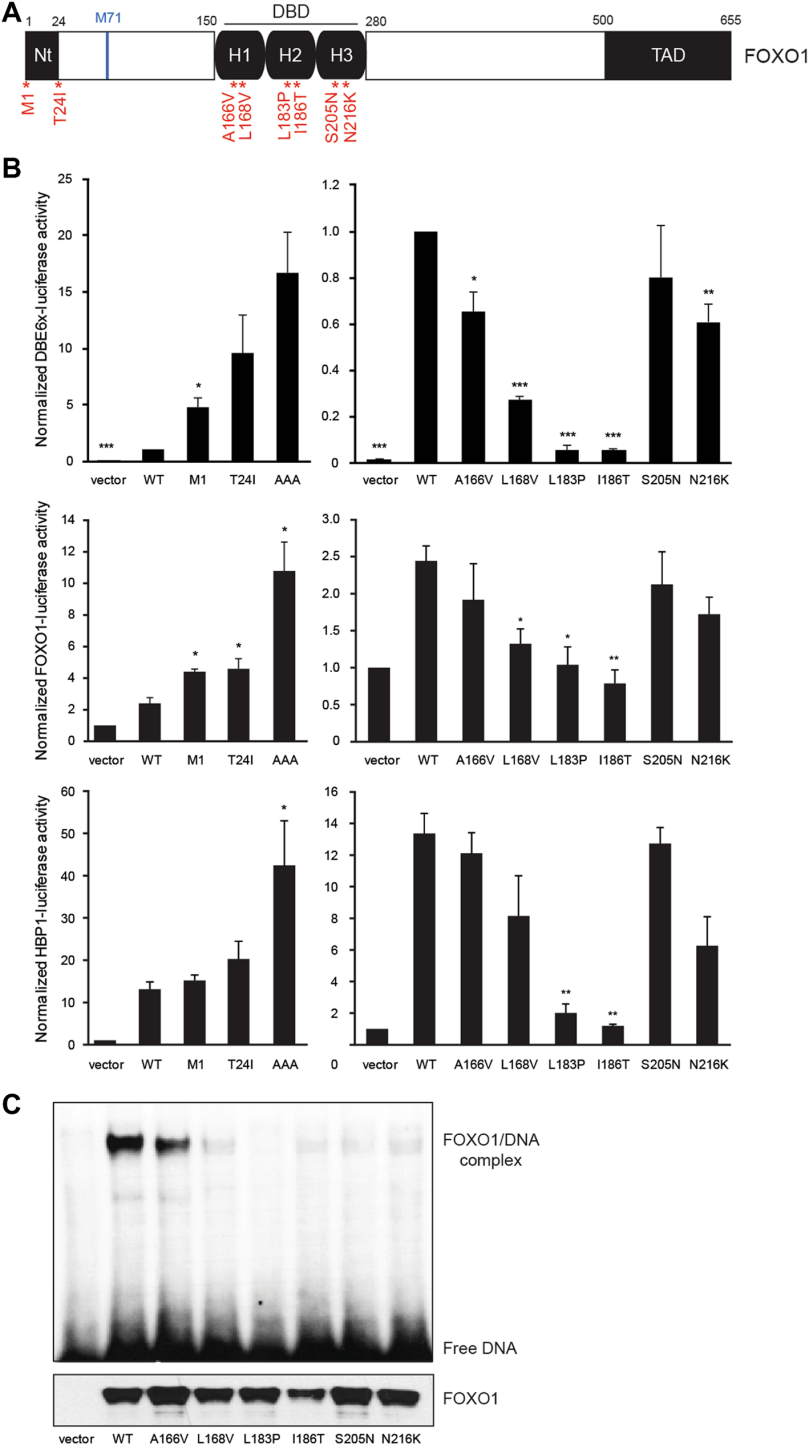
Transcriptional activity and DNA binding of FOXO1 Nt and DBD mutants. (**A**) Schematic structure of the human FOXO1 protein and the studied point mutations. (**B**) HEK293T cells were co-transfected with wild-type or mutated pCMV-FOXO1, luciferase reporter (DBE6x, *FOXO1* promoter, *HBP1* promoter) and the pEF-β-galactosidase vector as internal control. After 24 h of transfection, cells were lysed to measure the luminescence and the β-galactosidase activity. Average of 3 independent experiments is shown with standard error of the mean. Statistical analysis was calculated according to bilateral Student t-test (*, *p* < 0.05; **, *p* < 0.01; ***, *p* < 0.001). (**C**) Electrophoretic mobility shift assays (EMSA) were performed using nuclear extracts from HEK293T cells transfected with wild-type or mutated pCMV-FOXO1. A ^32^P-labeled oligonucleotide probe containing the FOXO consensus DNA-binding sequence (GTAAACA) was added to each nuclear extract. In addition, expression of FOXO1 was analyzed by western blot with an anti-FOXO1 antibody. Original unprocessed images are available in Supplementary Fig. S2.

In this study, we selected two Nt mutations and six DBD mutations among those initially reported by Trinh et al. in DLBCL and Pasqualucci et al. in follicular lymphoma based on amino acid conservation and predicted damage^8,11^ (Fig. 1A and Table 1). M1? and T24I mutants are the most frequent FOXO1 mutations in B-cell lymphoma^6^. As the M1? mutant produces a shift to the next start codon at position M71, both M1? and T24I mutants result in a loss of the AKT phosphorylation site Thr24, which is required for 14–3–3 protein binding and nuclear export^1^.

**Table 1:** Summary of molecular impact of FOXO1 DBD mutations.

Position	A166V	L168V	L183P	I186T	S205N	N216K
Forkhead domain localization	α-Helix 1	α-Helix 1	α-Helix 2	α-Helix 2	α-Helix 3	α-Helix 3
Amino acid conservation among FOXO family members	Yes	Yes	Yes	Yes	Yes	Yes
Predicted damage (mutationassessor.org)	Low	High	High	High	Low	High
DBE transcriptional activity (significant decreased compared to WT)	*	***	***	***	ns	**
DNA affinity (significant decreased compared to WT)	ns	ns	**	*	*	**

Statistical analysis was calculated according to bilateral Student t-test (*ns,* non-significant; *, *p* < 0.05; **, *p* < 0.01; ***, *p* < 0.001).

To determine the level of transcriptional activity of the different FOXO1 mutants, we carried out luciferase reporter assays with a reporter construct containing six motifs of the FOXO DNA-binding element GTAAACAA (DBE6x) upstream of the firefly luciferase gene and a minimal promoter. Expression of the FOXO1 mutants located in the DBD decreased the luciferase activity, especially L183P and I186T (Fig. 1B). By contrast, the FOXO1 Nt mutants M1? and T24I (and S22P, data not shown) increased the transcriptional activity by approximatively 5 and tenfold, respectively, compared to wild-type FOXO1. As a positive control, we included the AAA mutant, in which the three AKT phosphorylation sites (Thr24, Ser256, Ser319) are mutated into alanines^1^. In parallel, we corroborated these results by using two other reporter constructs, containing the gene promoters of *FOXO1* and *HBP1*, both of which were previously shown to recruit FOXO1 (Fig. 1B)^16,17^.

To investigate whether the loss of transcriptional activity of the DBD mutants resulted from a loss of DNA binding, we performed an electrophoretic mobility shift assay (EMSA) (Fig. 1C). FOXO1/DNA interaction was strongly reduced by most DBD mutations.

In conclusion, our results showed that DBD mutations are associated with a decreased transcriptional activity and DNA-binding ability.

### α-Helix 3 of the forkhead domain is crucial for FOXO1 transcriptional activity

**Figure 2:**
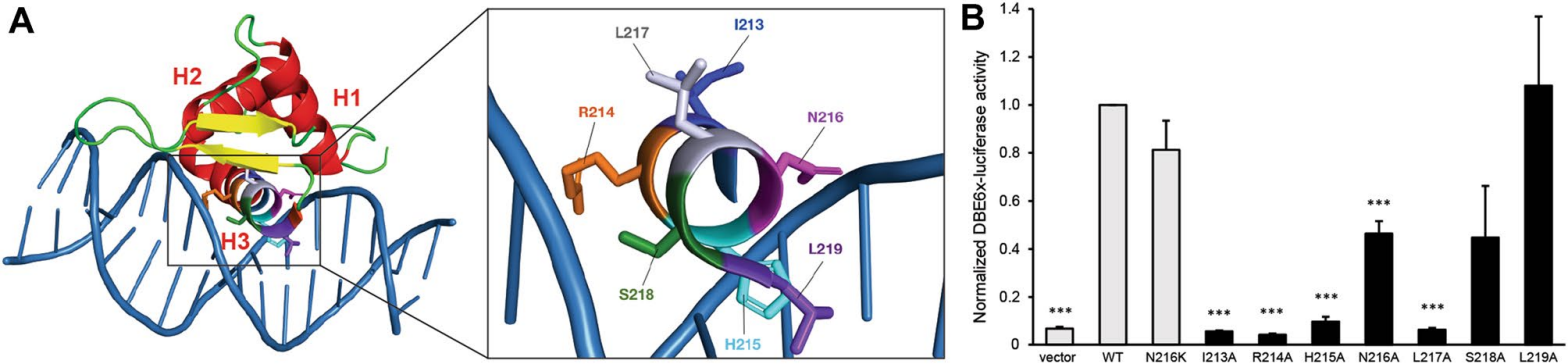
Alanine scanning mutagenesis of the α-helix 3 of FOXO1. (**A**) The 3D structure of the FOXO1 forkhead domain with the DNA double helix was visualized using PyMOL (PDB ID: 3COA). (**B**) HEK293T cells were co-transfected with wild-type or mutated pCMV-FOXO1, DBE6x-luciferase reporter and the pEF-β-galactosidase vector as internal control. The luminescence and the β-galactosidase activity were measured after 24 h. Average of 3 independent experiments is shown with standard error of the mean. Statistical analysis was calculated according to bilateral Student t-test (*, *p* < 0.05; **, *p* < 0.01; ***, *p* < 0.001).

To evaluate whether other substitutions could interfere with FOXO1 transcriptional activity, we mutated seven amino acids of the α-helix 3, which is directly inserted into the DNA major groove (Fig. 2A). This highly conserved sequence is considered as the main contact surface with the DNA-binding element (DBE) of FOXO1^18^. The luciferase assay with the DBE6x-luciferase reporter vector showed a significant decrease in luciferase activity for the I213A, R214A, H215A, N216A and L217A mutants (Fig. 2B). These data indicated that additional DBD residues are critical for FOXO1 function, confirming the importance of the α-helix 3.

### FOXO1 DBD mutants maintain non-transcriptional features

**Figure 3:**
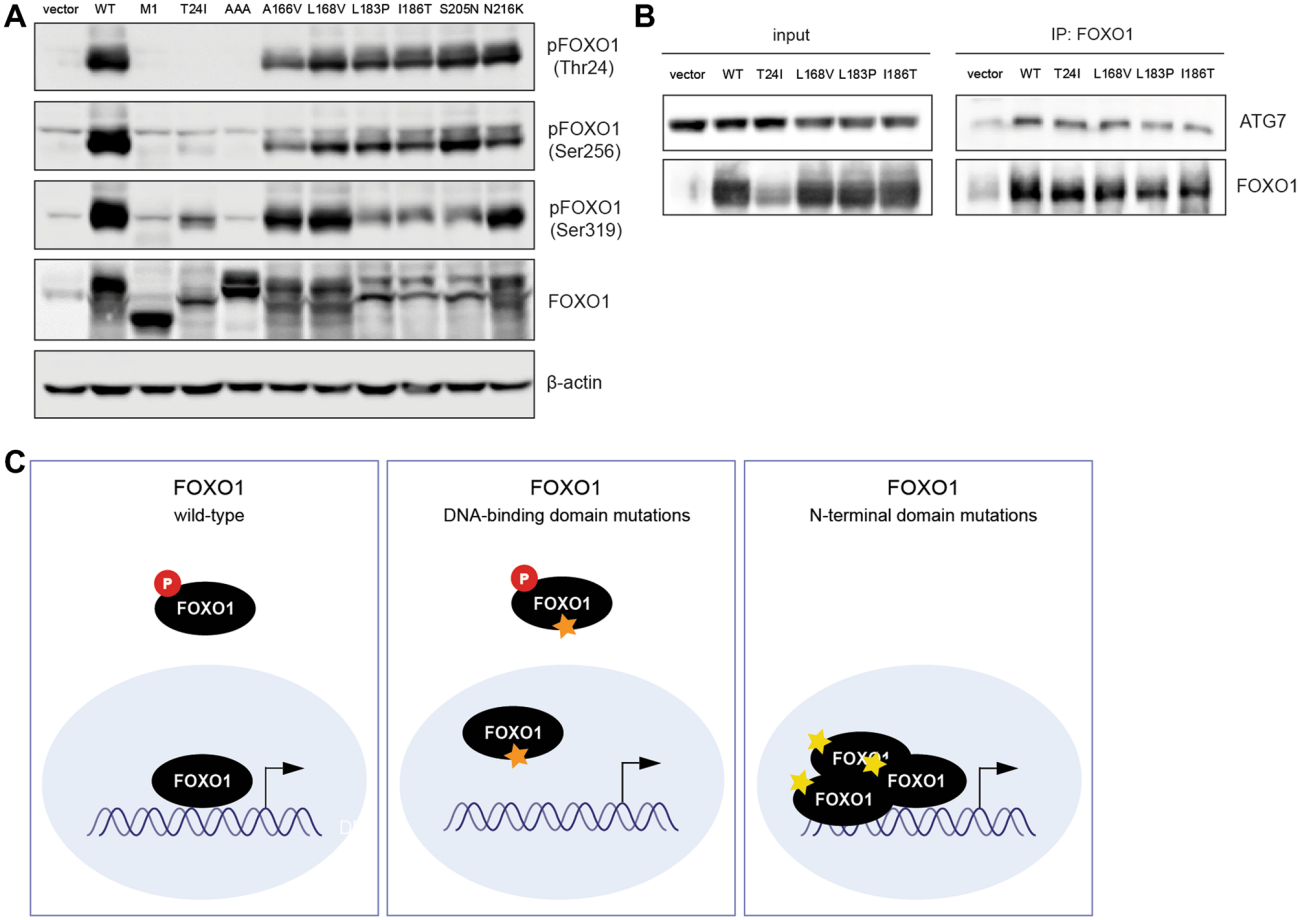
Expression of FOXO1 mutants, phosphorylation and interaction with ATG7. (**A**) HEK293T cells were transfected with wild-type or mutated pCMV-FOXO1 before protein extraction. Total protein lysates were analyzed by western blot with anti-phospho-FOXO1 (phospho-Thr24, phospho-Ser256 and phospho-Ser319), anti-FOXO1 and anti-β-actin antibodies. (**B**) HEK293T cells were transfected with wild-type or mutated pCMV-FOXO1 before protein extraction. FOXO1 was immunoprecipitated with anti-FOXO1 antibody and analyzed by western blot with anti-FOXO1 and anti-ATG7 antibodies. Original unprocessed images are available in Supplementary Fig. S2. (**C**) Schematic conclusion of cellular and molecular mechanisms of FOXO1 DBD versus Nt mutations.

To further analyze the impact of DBD mutations on FOXO1 functions, we studied their phosphorylation profile. DBD mutants were phosphorylated on the three AKT phosphorylation sites like wild-type FOXO1 (Fig. 3A). By contrast, we observed the expected loss of phosphorylation of Thr24 by the M1? and T24I mutants. Surprisingly, they also presented a decreased phosphorylation of Ser256 and Ser319.

FOXO1 interacts with multiple proteins, among which ATG7 is of particular interest, as it contributes to the regulation of autophagy, a non-transcriptional function of FOXO^19^. We showed by co-immunoprecipitation that the ability of FOXO1 L168V, L183P and I186T to bind ATG7 was not affected (Fig. 3B). These results suggested that the non-transcriptional properties of FOXO1 are not impacted by mutations in the DNA-binding domain.

## Discussion

While recent reports have focused on Nt mutations, we demonstrated in this study that mutations of the forkhead DNA-binding domain of FOXO1 confer a loss of function by preventing DNA binding and reducing its transcriptional activity. Most DBD mutations are located in the helical structures of the forkhead domain and the mutated amino acids are highly conserved among FOX proteins. In particular, L183 and N216 were suggested to contact DNA^18^. Accordingly, the L183P mutant presented a complete loss of DNA binding and transcriptional activity. Similar results were obtained with the I186T substitution, which is also located in the α-helix 2 but does not contact DNA. Other variants, L168V, S205N and N216K, had a much reduced ability to bind DNA in vitro, but retained some transcriptional activity. The formation of a large protein complex on gene promoters in luciferase assays may partially compensate for the lower affinity of these FOXO1 mutants, observed in EMSA.

Since the initiation of the present study, additional DBD mutants were reported in the literature and in databases (see Supplementary Fig. S1 for an updated list of *FOXO1* variants in B-cell malignancies). These mutations are scattered throughout the DBD domain, with only few recurrent positions such as Ser152 (7 cases), Ala166 (10 cases) and Ser205 (9 cases), as one would expected if they confer a loss of DBD function.

Furthermore, we searched for additional residues that could be essential for FOXO1 activity by introducing alanine substitutions in the α-helix 3. Indeed, four mutants out of seven presented a complete loss of function. H215 had already been identified as a key amino acid for FOXO1 activity as its mutation in arginine (H215R) disrupted DNA binding^20^. Moreover, crystal structures showed direct base-specific contact between side chains of residues N211 and H215 with promoters regulated by FOXO1^18,21,22^. However, the orientation of I213 and L217 side chains suggest that these amino acids are not in direct contact with DNA, despite a complete loss of activity when mutated in alanine. Hence, we can not predict damage on the sole basis of structural criteria. To our knowledge, only the N216K variant has been identified in non-Hodgkin lymphoma^8,11^. In addition, the R214C, R214H, S218F and L219I mutations have been reported in non-hematological malignancies in the COSMIC database. Of particular interest, substitutions of the R214 residue has been reported in 11 cases.

Then, we suggested that DBD mutants might still participate to cellular functions independently of DNA binding. Our data showed that FOXO1 DBD mutants are still regulated by AKT such as wild-type FOXO1 but can not bind DNA, whereas FOXO1 Nt mutants are devoid of AKT phosphorylation, leading to an increased transcriptional activity (Fig. 3C). Moreover, we observed that FOXO1 L183P and I186T remained able to co-immunoprecipitate with ATG7, a protein involved in the autophagy process^19^. Interestingly, the classical loss-of-function alterations seen in tumor-suppressor genes, such as frameshifts, premature stop codons or gene deletions, were rarely reported in the *FOXO1* gene in B-cell lymphoma (Supplementary Fig. S1). Therefore, we hypothesize that DBD mutations may selectively disrupt DNA-dependent tumor-suppressor activities while preserving other FOXO1 functions beneficial to B lymphoma cells.

Altogether, our findings shed light on a new type of *FOXO1* mutations in B-cell malignancies. Their impact on lymphoma development must be investigate in future experiments. Regarding the opposite effects of DBD and Nt mutants on FOXO1 activity, we speculate that these alterations could appear at different time points of B-cell differentiation or at different germinal center reaction steps, given the complex role of FOXO1 during the B-cell development^23^. Alternatively, the two types of mutations may occur at different points of the treatment course. This hypothesis is supported by the observation that FOXO1 activation decreases CD20 expression and the sensitivity to rituximab^24,25^. However, the *FOXO1* mutation profile does not seem to differ between DLBCL at diagnosis and after relapse^26^. Nevertheless, the number of patients was too small to draw a definitive conclusion. A more detailed analysis of patients classified according to the type of mutation is therefore needed. However, this will require larger cohorts of patients with detailed sequencing data, which are not available yet.

In conclusion, our results suggest that FOXO1 mutations in lymphoma may have opposite functional consequences on its transcriptional activity, further illustrating the complex role of this transcription factor in cancer.

## Materials and methods

### Cell culture, reagents and vectors

HEK293T cells (ATCC, Manassas, VA, USA) were cultured in Dulbecco's modified Eagle's medium (DMEM, Gibco, Grand Island, NY, USA) supplemented with 10% fetal bovine serum. Anti-phospho-FOXO1 (anti-Thr24 (#9464), anti-Ser256 (#9461), anti-Ser319 (#2486)), anti-FOXO1 (#2880) and anti-ATG7 (#2631) antibodies were purchased from Cell Signaling Technology (Danvers, MA, USA) and the anti-β-actin antibody (#A5441) from Sigma (Saint-Louis, MO, USA). We obtained human wild-type FOXO1 cloned in pCMV6-XL4 (OriGene, Rockville, MD, USA). Point mutations were introduced by site-directed mutagenesis using the QuickChange XL-II kit (Stratagene, La Jolia, CA, USA) according to the manufacturer’s protocol.

### Luciferase assay

The pGL3-basic luciferase reporter vectors (pGL3-DBE6x, pGL3-FOXO1 and pGL3-HBP1, previously described^16,17^) were co-transfected with a wild-type or mutated pCMV6-XL4-FOXO1 construct and the pEF1-β-galactosidase (Invitrogen, Carlsbad, CA, USA) as internal control according to the calcium phosphate protocol (0.150 µg of each plasmid in a final volume of 100 µl). The luciferase activity was monitored in cell lysates using a GLOMAX instrument (Promega) and normalized with the β-galactosidase activity as described^27^.

### Electrophoretic mobility shift assay

Electrophoretic mobility shift assays (EMSA) were performed using nuclear extracts, as described^28^. Nuclear extracts (corresponding to 4 µg of total proteins) were incubated on ice for 15 min in a binding buffer (10 mM Tris/HCl pH 8, 150 mM KCl, 0.5 mM EDTA, 0.1% Triton X-100, 12.5% glycerol, 0.2 mM DTT) supplemented with 1 µg poly(dI-dC) (Invitrogen). A ^32^P-labeled oligonucleotide probe (0.5 pmol) containing the FOXO consensus DNA-binding sequence (GTAAACA) was added to each sample on ice for 15 min. A 6% polyacrylamide gel was beforehand equilibrated without any sample for 2 h at 20 mA using 0.25 × TBE (22.25 mM Tris base pH 8, 22.25 mM boric acid, 0.5 mM EDTA) as running buffer. Samples were then loaded on the polyacrylamide gel for 2 h at 30 mA. The gel was dried for 1 h at 70 °C and the radioactivity was visualized by phosphorimaging (Thyphoon TRIO, GE Heathcare).

### Protein extraction, co-immunoprecipitation and western blot

Following transfection, cells were washed in PBS before lysis in buffer (25 mM Tris/HCl pH 7.4, 150 mM NaCl, 6 mM EDTA, 10% glycerol, 1% Triton X-100) containing protease inhibitors (1 mM sodium orthovanadate, 1 mM Pefabloc and 1 μg/ml aprotinin) for 20 min on ice. Extracts were cleared by high speed centrifugation and protein concentration was measured using the BCA Protein Assay Kit (Thermo Fisher Scientific, Whaltham, MA, USA). For co-immunoprecipitation, cells were lysed in RIPA buffer (50 mM Tris/HCl pH 7.4, 150 mM NaCl, 1 mM EDTA, 0.25% sodium deoxycholate, 1% NP-40) containing protease inhibitors and 3 mg of proteins were precipitated overnight at 4 °C with anti-FOXO1 antibody. Immunoprecipitates were then incubated 1h30 at 4 °C with protein A/G-coupled beads (Thermo Fisher, #53133) and washed 3 times before boiling. Samples were analyzed by western blot, using 8% polyacrylamide gels and polyvinylidene difluoride membranes, which were then blocked and incubated with the indicated antibodies. Chemiluminescence was visualized by the Fusion Solo S (Vilber, France).

## Supplementary Information

Supplementary Information.
