# Supplementary material for: FOXO1 forkhead domain mutants in B-cell lymphoma lack transcriptional activity
Source: Sci Rep. 2022 Jan 25;12:1309. doi: 10.1038/s41598-022-05334-4 (PMC8789783; doi:10.1038/s41598-022-05334-4)

Supplementary Information

FOXO1 forkhead domain mutants in B-cell lymphoma lack transcriptional activity

Ariane Sablon*[1], Emeline Bollaert*[1], Constance Pirson[1], Amélie I. Velghe[1], Jean-Baptiste Demoulin[1]
[1] de Duve Institute, Université Catholique de Louvain, Brussels, Belgium.
*These authors contributed equally to this work.

Supplementary Figure S1. FOXO1 forkhead domain mutations in B-cell malignancies. Mutations reported in the literature, in COSMIC and in cBioPortal (December 6, 2021). Studied variants are highlighted in gray. CLL: chronic lymphocytic leukemia; DLBCL: diffuse large B-cell lymphoma; BL: Burkitt lymphoma; CNSL: central nervous system lymphoma; FL: follicular lymphoma; PTLD: post-transplant lymphoproliferative disorder; MZL: marginal zone lymphoma.

CDS mutations	AA mutations	Lymphomas	Counts	References
c.455G>A	p.S152N	CLL	1	COSM7208266
c.456C>A	p.S152R	DLBCL (2), BL (2), CNSL (1)<br>DLBCL	5<br>1	COSM9213941<br>Trinh et al, 2013
c.457A>G	p.S153G	DLBCL	1	Reddy et al, 2017
c.459C>G	p.S153R	DLBCL	1	Trinh et al, 2013
c.486C>G	p.N162K	DLBCL<br>DLBCL	1<br>1	COSM8438787<br>cBioPortal TCGA-VB-A8QN
c.496_588del	p.A166_Y196del	DLBCL	1	COSM9863544
c.496G>A	p.A166T	DLBCL	1	Reddy et al, 2017
c.497C>T	p.A166V	DLBCL<br>DLBCL<br>DLBCL	4<br>2<br>1	COSM9866300<br>Trinh et al, 2013<br>Reddy et al, 2017
c.497C>G	p.A166G	DLBCL	1	Trinh et al, 2013
c.502C>G	p.L168V	DLBCL	1	Trinh et al, 2013
c.511A>G	p.K171E	DLBCL	1	Trinh et al, 2013
c.514G>A	p.A172T	DLBCL	1	Reddy et al, 2017
ns	p.I173M	BL	1	Zhou et al, 2019
c.524G>A	p.S175N	DLBCL	1	COSM6954168
c.524G>C	p.S175T	DLBCL	1	Dubois et al, 2016
ns	p.I175R	BL	1	Zhou et al, 2019
c.536_547del	p.K179_L183delinsM	DLBCL	1	COSM9863413
c.540_626delinsA	p.L181Efs*7	DLBCL	1	COSM9862360
c.548T>C	p.L183P	DLBCL	1	Trinh et al, 2013
c.557T>C	p.I186T	DLBCL	1	Trinh et al, 2013
ns	p.I186S	BL	1	Zhou et al, 2019
c.567G>A	p.W189*	FL	1	COSM1316591
c.568A>G	p.M190V	DLBCL	1	Reddy et al, 2017
c.578G>A	p.S193N	DLBCL	1	COSM7338533
c.579C>A	p.S193R	FL	1	COSM220649 (Morin et al, 2011)
c.581T>C	p.V194A	BL	2	COSM9213939
c.588C>G	p.Y196*	DLBCL	1	COSM9867401
c.592A>T	p.K198*	DLBCL	1	COSM9868106
c.595G>C	p.D199H	PTLD	1	COSM7338768
c.598A>T	p.K200*	PTLD	1	COSM7338760
c.608G>A	p.S203N	FL	1	COSM220643 (Morin et al, 2011)
c.609C>A	p.S203R	PTLD	1	COSM7338756
c.611_614delinsGCTA	p.N204_S205delinsSY	DLBCL	1	COSM6986743
c.612C>G	p.N204K	MZL	1	COSM9264648
c.614G>A	p.S205N	DLBCL<br>DLBCL	3<br>1	COSM5651478 (Morin et al, 2016)<br>Trinh et al, 2013
c.614G>C	p.S205T	DLBCL (1), PTLD (1)<br>SU-DHL5 cell line<br>DLBCL<br>BL	2<br>1<br>1<br>1	COSM7338758<br>Trinh et al, 2013<br>Dubois et al, 2016<br>Zhou et al, 2019
ns	p.S206L	BL	1	Zhou et al, 2019
ns	p.A207P	BL	1	Zhou et al, 2019
c.648T>A	p.N216K	DLBCL<br>FL	1<br>1	COSM4170502<br>Pasqualucci et al, 2014

References:

- Morin, R.D., et al., Frequent mutation of histone-modifying genes in non-Hodgkin lymphoma. Nature, 2011. 476(7360): p. 298-303.
- Trinh, D.L., et al., Analysis of FOXO1 mutations in diffuse large B-cell lymphoma. Blood, 2013. 121(18): p. 3666-74.
- Pasqualucci, L., et al., Genetics of follicular lymphoma transformation. Cell Rep, 2014. 6(1): p. 130-40.
- Dubois, S., et al., Next-Generation Sequencing in Diffuse Large B-Cell Lymphoma Highlights Molecular Divergence and Therapeutic Opportunities: a LYSA Study. Clin Cancer Res, 2016. 22(12): p. 2919-28.
- Morin, R.D., et al., Genetic Landscapes of Relapsed and Refractory Diffuse Large B-Cell Lymphomas. Clin Cancer Res, 2016. 22(9): p. 2290-300.
- Reddy, A., et al., Genetic and Functional Drivers of Diffuse Large B Cell Lymphoma. Cell, 2017. 171(2): p. 481-494 e15.
- Zhou, P., et al., Sporadic and endemic Burkitt lymphoma have frequent FOXO1 mutations but distinct hotspots in the AKT recognition motif. Blood Adv, 2019. 3(14): p. 2118-2127.

Supplementary Figure S2. Original images of Figure 1C, 3A, 3B.
Unprocessed images of EMSA and western blots.

Original images of Figure 1C.

EMSA. Image detected with a phosphorimager screen overnight.

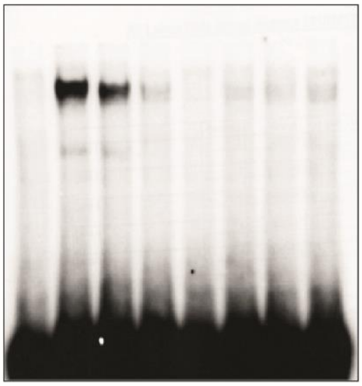

Western blot of FOXO1 expression. Signal detected with "Amersham Hyperfilm ECL". The whole piece of film is shown.

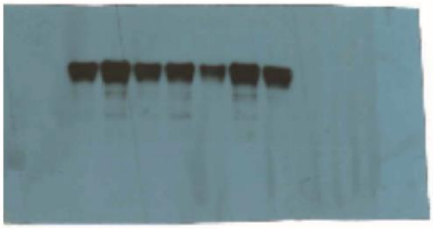

Original images of Figure 3A.

Western blots of phospho- and total FOXO1. Signal detected with FUSION Solo S (Vilber).

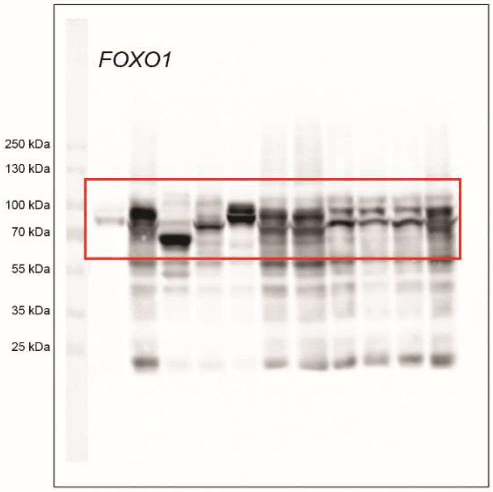

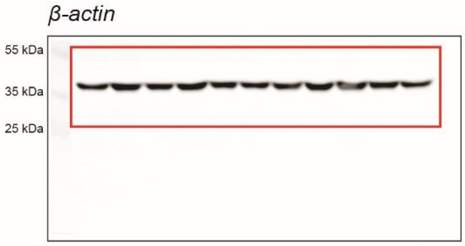

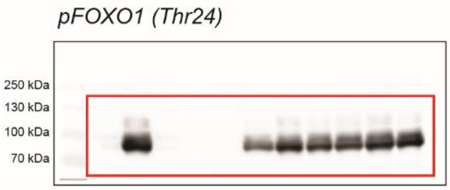

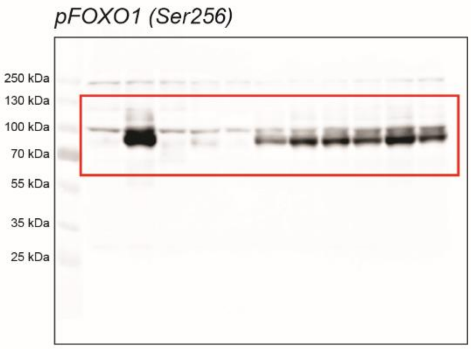

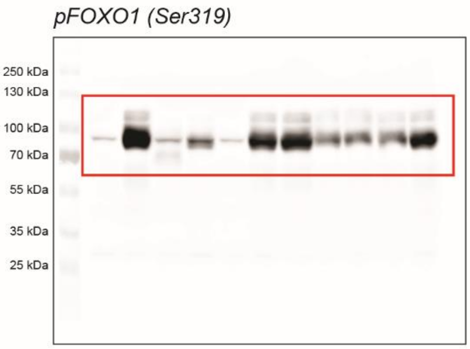

Original images of Figure 3B.

Western blot of phospho- and total FOXO1. Signal detected with FUSION Solo S (Vilber).

FOXO1 (low exposure)

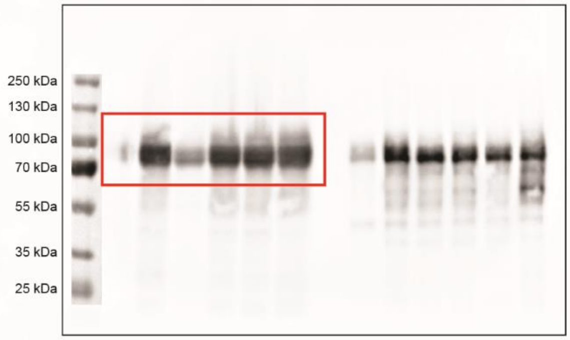

FOXO1 (high exposure)

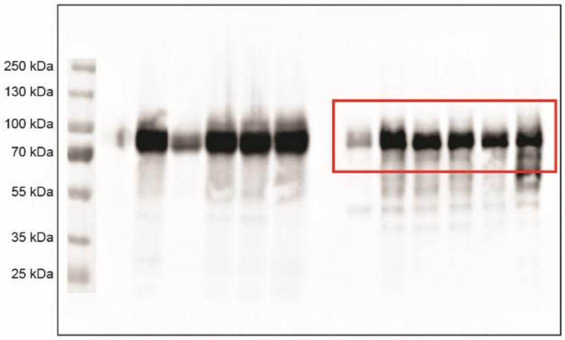

ATG7 (low exposure)

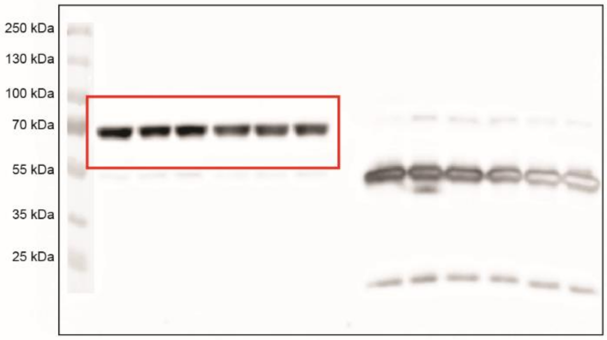

ATG7 (high exposure)

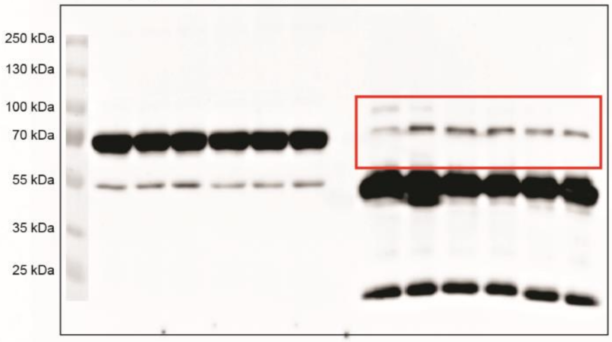

Supplement: Supplementary file 1 — Supplementary Information. [file 41598_2022_5334_MOESM1_ESM.pdf]
